# Phylogeography of *Amygdalus mongolica* in relation to Quaternary climatic aridification and oscillations in northwestern China

**DOI:** 10.7717/peerj.13345

**Published:** 2022-04-29

**Authors:** Lin Zhang, Fangfang Sun, Songmei Ma, Chuncheng Wang, Bo Wei, Yunling Zhang

**Affiliations:** 1 Shihezi University, Xinjiang Production and Construction Corps Key Laboratory of Oasis Town and Mountain-basin System Ecology, College of Science, Shihezi, Xinjiang, China; 2 Shihezi University, Xinjiang Production and Construction Corps Key Laboratory of Oasis Town and Mountain-basin System Ecology, College of Life Sciences, Shihezi, Xinjiang, China; 3 Institute of Geographic Sciences and Natural Resources Research, Beijing, China; 4 General grassland station of Xinjiang, Urumqi, Xingjiang, China

**Keywords:** *Amygdalus mongolica*, Allopatric differentiation, Postglacial expansion, Genetic conservation, Northwestern China

## Abstract

Quaternary period geological events and climatic oscillations significantly affect the geographic structure and genetic diversity of species distribution in arid northwestern China. *Amygdalus mongolica* is a relict and endangered shrub that occurs primarily in arid areas of northwestern China. Based on variation patterns present at three cpDNA regions (*psb*K-*psb*I, *trn*L-*trn*F and *trn*V) and in one nDNA sequence (ITS1-ITS4) in 174 individuals representing 15 populations, the spatial genetic structure and demographic history of *A. mongolica* was examined across its entire geographic range. The 17 different haplotypes and 10 ribotypes showed two lineages, distributed across the Western (Mazong Mountains, Hexi Corridor, and Alxa Left Banner) and Eastern regions (Urad Houqi, Yinshan Mountains, Urad Zhongqi, and Daqing Mountains) according to the median-joining network and the BI (Bayesian inference) and ML (Maximum likelihood) trees. AMOVA analysis demonstrated that over 65% of the observed genetic variation was related to this lineage split. The expansions of the Ulanbuhe and Tengger deserts and the eastward extension of the Yinshan Mountains since the Quaternary period likely interrupted gene flow and triggered the observed divergence in the two allopatric regions; arid landscape fragmentation accompanied by local environmental heterogeneity further increased local adaptive differentiation between the Western and Eastern groups. Based on the evidence from phylogeographical patterns and the distribution of genetic variation, *A. mongolica* distributed in the eastern and western regions are speculated to have experienced eastward migration along the southern slopes of the Lang Mountains and westward migration along the margins of the Ulanbuhe and Tengger deserts to the Hexi Corridor, respectively. For setting a conservation management plan, it is recommended that the south slopes of the Lang Mountains and northern Helan Mountains be identified as the two primary conservation areas, as they have high genetic variation and habitats that are more suitable.

## Introduction

Climatic oscillations, especially those that occurred in the Pleistocene glacial-interglacial cycles during the Quaternary period, shaped contraction and expansion patterns of species, and undoubtedly left genetic signatures in extant populations (Arbogast, 2001; Hewitt, 1996). Many plant phylogeographic studies using the combined approach of molecular data and paleoclimatic evidence have found that Pleistocene glaciations had a profound influence on the genetic diversity, population structure, and evolutionary history of species throughout the Northern Hemisphere (Hewitt, 2000; Riccioni et al., 2019). In the arid region of northwest China, although pollen records and paleodata show no evidence of glaciation (Shi, 2006), Quaternary climatic shocks have also profoundly affected the local plant growth, during the Last Glacial Maximum, the current coniferous and deciduous forests were replaced by steppe and even desert vegetation in northern and northwestern China (Harrison, G. Yu & Prentice, 2001; Ni et al., 2006; Yu et al., 2000). Aridification occurred in northwest China due to the uplifts of the Tibet Plateau and the difficulty of water vapor reaching inland areas, and further intensified as desert conditions began to emerge during the late Miocene (Zhang & Jiang, 1992). At the same time, extremely arid climates and geological events created fragmented landscapes in northwestern China, potentially having led to the reduction of suitable plant habitat in these areas (Jia & Zhang, 2021; Li et al., 2019; Qiu et al., 2017). The fragmented landscape in arid northwestern China during the Quaternary period restricted gene flow between different geographical populations, and has played significant roles in determining divergence and diversification of local desert plants in isolated and allopatric regions. Currently, there is significant regional divergence in xerophytic *Tugarinovia mongolica* from Inner Mongolia due to habitat fragmentation following enhanced aridification (Zhao et al., 2019a). Intraspecific differentiation and the deeply shaped genetic structure for *Gymnocarpos przewalskii* and *Lagochilus ilicifolius* were primarily caused by variation of desert habitats during the Pleistocene (Gao, Meng & Zhang, 2014; Zhang, Wang & Jia, 2020). In contrast, xerophytic plants such as *Zygophyllum xanthoxylon* and *Clematis songorica* appeared to adapt to the changes in aridity, and no evidence of genetic divergence among different populations was detected (Shi & Zhang, 2015; Zhang et al., 2013). Several refugia for several local desert species have been revealed in fragmented and arid areas in the Tarim Basin, Tianshan Mountains, Hami Basin, Helan Mountains and northwestern areas of Inner Mongolia in northwestern China, and evidence of post-glacial expansion following glacial periods has been detected (Shi & Zhang, 2015; Xu & Zhang, 2015; Zeng et al., 2018). The arid Tianshan Mountains have greatly affected many plants genetic structure, such as *Clematis sibirica* of a woody perennial vine growing primarily under conifer forests (Zhang & Zhang, 2012) and *Delphinium naviculare* growing at forest edges and in grassy slopes (Zhang et al., 2013); the Helan Mountains likely acted as the center of diversification for *Lagochilus ilicifolius* (Meng & Zhang, 2011) and were inferred as the refugial locations for *Zygophyllum xanthoxylon* (Shi & Zhang, 2015).

*Amygdalus mongolica* is a xerophitic desert shrub that ranges from the Mazong Mountains in the northernmost Gansu, through the Hexi Corridor and Alxa Left Banner to the Urad Houqi, Yinshan Mountains, Urad Zhongqi, and Daqing Mountains in northeastern Inner Mongolia in northwestern China (Fu & Chin, 1992). The area for this study was concentrated in areas of severe aridity, characterized by low annual precipitation and high evaporation (Ma et al., 2015), where altitude was greater than 1,000 m and the terrain was mainly plateaus (Alxa Plateau and Ordos Plateau) and desert landscapes (Badain Jaran Desert, Tengger Desert, Ulan Buhe Desert and Kubuqi desert). The Ordos Plateau is surrounded by the Yin Mountains in an east–west direction with many deep valleys, and the Helan Mountains in a south-north direction that has many valleys and steep terrain. *A. mongolica*, as a Tertiary Miocene relic plant of the Ancient Mediterranean, offers an optimal case for investigating the evolutionary processes in response to the Quaternary climate oscillations and aridification in arid northwestern China (Fu & Chin, 1992). Moreover, it is endangered (listed on the China Species Red List) and is a protected plant of third conservation priority (Fu & Chin, 1992). Because of this, the genetic structure of the *A. mongolica* population has high importance, for understanding both the origin and evolution of plants in the Chinese northwestern desert, as well as for plant protection and conservation. In a previous study by our group, the presence of two chloroplast intergenic spacers (*psb*K-*psb*I and *trn*L-*trn*F) supported two fragmented geographic groups among different populations of *A. mongolica* (Ma et al., 2019). A large number of shared haplotypes exist between the two geographic groups, however, likely because of incomplete lineage sorting; the limited polymorphic fragments were deficient for studying population dynamics, genetic structure, and conservation of species genetic diversity.

Previous studies have shown that a variety of genes, such as maternally inherited chloroplast DNA (cpDNA) markers in combination with biparentally inherited nuclear DNA (nDNA) markers, show an integral view for identifying the relationships and genetic structure among taxonomic groups and populations (Li et al., 2019). Here, multiple methods of population genetic analysis and landscape genetic analysis were used along with a least-cost path (LCP) analysis to investigate the genetic structure and demographic history of 15 natural populations of *A. mongolica* in northwestern China based on three highly polymorphic chloroplast DNA fragments (*psb*K-*psb*I, *trn*L-*trn*F and *trn*V) and a nuclear marker (ITS1-ITS4). This study aimed to assess the spatial genetic structure and intraspecific differentiation and detect whether incomplete lineage sorting exists in *A. mongolica* populations in northwestern China according to cpDNA and nDNA sequences variation, inference of phylogeographic and demographic history of *A. mongolica* populations during Quaternary climate fluctuations and desert expansion was also performed, along with comprehensive identification of potential conservation areas for *A. mongolica* populations.

## Material and Methods

### Sample collection

**Figure 1:**
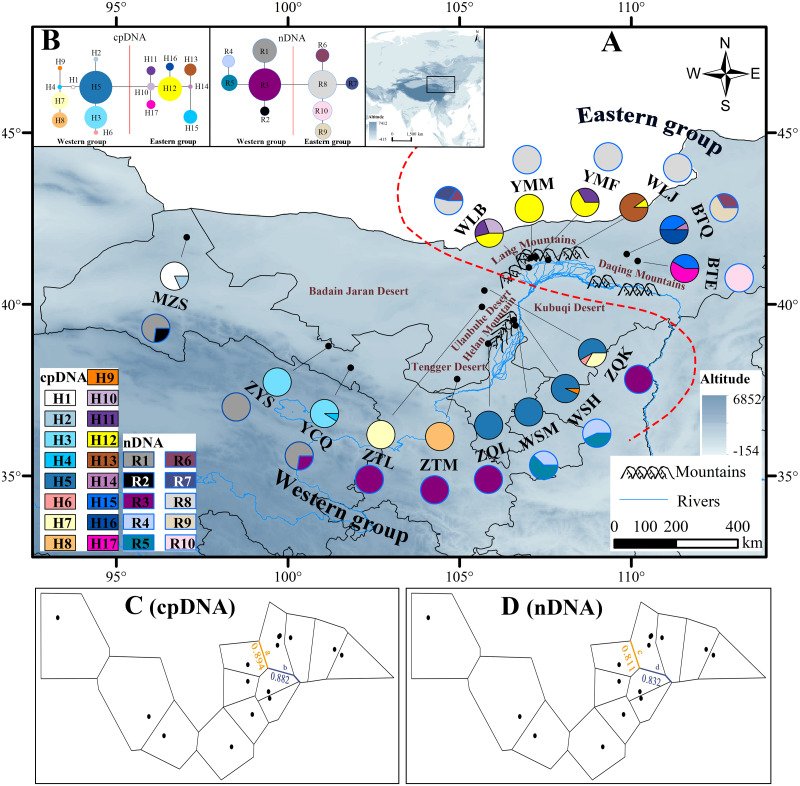
Map of the sampling sites, geographical distribution of the chlorotypes and ribotypes, the phylogenetic network and genetic barriers among populations of *Amygdalus mongolica*. (A) Sampling localities and geographic distribution of 17 cpDNA haplotypes (labelled as H1–H17) and 10 nDNA ribotypes (labelled as R1–R10), identified from 15 populations of *A*. *mongolica* in Northwest China. Pie graphs reflect the frequency of each haplotype (the black pie) and each ribotype (the red pie), the population codes listed in Table 1. (B) The median-joining network for the 17 haplotypes and 10 ribotypes. The sizes of the circles in the network are proportional to haplotype/ribotype frequencies. Branch lengths are roughly proportional to the number of mutation steps between haplotypes/ribotypes and nodes. (C, D) Genetic barriers to chlorotypes and ribotypes between different sampling areas, respectively.

A total of 174 individuals of *A. mongolica* from 15 natural populations were investigated (Table 1) throughout its geographic distribution in northwestern China (Fig. 1A). The details of sampling populations are described in our previous study (Ma et al., 2019), including three from Gansu Province and twelve from Inner Mongolia (Table 1). Fresh young leaves were collected from 7–15 individuals in each population. Individual plants were sampled at least 10 m apart to avoid sampling close relatives and to ensure a robust sampling. The leaves were dried on silica gel and stored at 4 °C until DNA extraction. The Provincial administrative boundary data of the study area were obtained from the Data Center of Resources and Environment Sciences, Chinese Academy of Sciences (http://www.resdc.cn/).

**Table 1:** Sample and genetic variation information for 15 populations of *Amygdalus mongolica*.

Population location/code	Lat/Lon	Sample size	cpDNA haplotype	*H*_d_ (± SD)	cpDNA *π* (± SD*10^−2^)
			nDNA ribotype	*R*_d_ (± SD)	nDNA *π* (± SD*10^−2^)
**Western group**		102		0.791 ± 0.036 0.720 ± 0.045	0.00683 ± 0.33 0.00591 ± 0.21
MZS	41.95°/97.05°	11	H1 (9), H2 (2) R1 (8), R2(3)	0.327 ± 0.15 0.436 ± 0.13	0.00842 ± 0.39 0.00341 ± 0.21
ZYS	38.78°/101.18°	11	H3 (11) R1 (11)	– –	– –
YCQ	38.15°/101.83°	11	H3(10), H4 (1) R1 (8), R3 (3)	0.439 ± 0.16 0.431 ± 0.01	0.00574 ± 0.22 0.00711 ± 0.30
ZTL	39.92°/105.65°	12	H7 (12) R3 (12)	– –	– –
ZQK	40.40°/105.72°	12	H5 (7), H6 (1), H7 (4) R3 (12)	0.534 ± 0.06 –	0.00962 ± 0.13 –
ZTM	37.82°/104.93°	12	H8 (12) R3 (12)	– –	– –
ZQL	38.85°/105.83°	11	H5 (11) R3 (11)	– –	– –
WSM	39.38°/106.63°	7	H5 (7) R4 (4), R5 (3)	– 0.509 ± 0.01	– 0.00683 ± 0.32
WSH	39.54°/106.58°	15	H5 (14), H9 (1) R4 (9), R5 (6)	0.417 ± 0.01 0.513 ± 0.008	0.00342 ± 0.23 0.00911 ± 0.39
**Eastern group**		72		0.804 ± 0.04 0.576 ± 0.01	0.00723 ± 0.24 0.00394 ± 0.19
WLB	41.07°/107.02°	13	H10 (4), H11 (3), H12 (6) R6 (2), R7 (4), R8 (7)	0.567 ± 0.13 0.676 ± 0.07	0.00544 ± 0.11 0.00922 ± 0.39
YMM	41.35°/107.08°	13	H12 (13) R8 (13)	– –	– –
YMF	41.34°/107.09°	12	H11 (4), H12 (8) R8 (12)	0.467 ± 0.132 –	0.00681 ± 0.32 –
WLJ	41.29°/107.58°	10	H12 (1), H13 (9) R8 (10)	0.410 ± 0.15 –	0.00652 ± 0.34 –
BTQ	40.92°/109.58°	12	H14 (1), H15 (5), H16 (6) R6 (4), R9 (8)	0.530 ± 0.076 0.513 ± 0.082	0.00682 ± 0.22 0.00612 ± 0.17
BTE	40.72°/109.90°	12	H15 (5), H17 (7) R10 (12)	0.447 ± 0.032 –	0.00211 ± 0.13 –

### Laboratory procedures

Total genomic DNA was extracted from approximately 100 mg of silica gel-dried leaf tissue using a modified 2 × CTAB protocol (Doyle, 1987). Three polymorphic chloroplast DNA intergenic spacers, *psb*K-*psb*I, *trn*L-*trn*F and *trn*V (Shaw et al., 2007) and one nDNA fragment, ITS1-ITS4 (White et al., 1990) were chosen for this study.

We used a 30 µL volume containing 20 mM dNTP, 2 mM MgCl_2_, 0.025 U mL^−1^ *Taq* polymerase and 1 pmol of each primer (Takara Co. Ltd., Beijing, China) to conduct the polymerase chain reaction (PCR) (Takara Co. Ltd., Beijing, China). The DNA amplification profile was 94 °C for 2 min, followed by 30 cycles of 94 °C for 30 s, annealing at 52 °C (*psb*K-*psb*I, *trn*L-*trn*F), 54 °C (*trn*V) and 55 °C (ITS1-ITS4) for 30 s, 72 °C for 90 s, and a final extension at 72 °C for 10 min. The processing method of all PCR products were previously described in Ma (Ma et al., 2015). Specifically, the study used a PCR product purification kit (Viogene, Sunnyvale, CA, USA) to purify from agarose gel (0.1–0.5%) and used the ABI 3730 DNA Analyzer to sequence on forward and reverse strands by BigDye terminator chemistry (Applied Biosystems, Foster City, CA, USA). For the heterozygous sites for nuclear genes after sequencing, using the Bayesian approach of PHASE 2.1 to infer haplotypes from genotype data (Stephen & Donnelly, 2003), and our study was corrected using default parameters in DnaSP 6.0 and verified in MEGA-X ver. 10.1.8. Sequences were aligned using CLUSTAL X1.83 (Thompson et al., 1997) and then manually used BioEdit 7.2.5 (Hall, 1999) for alignment. Each insertion/deletion in this study was treated as a single mutation event and encoded as substitutions in subsequent analyses (Simmons & Ochoterena, 2000).

### Genetic diversity and genetic structure analyses

Molecular parameters, containing cpDNA haplotype/nDNA ribotype diversity (*H*_d_, *R*_d_), and nucleotide diversity (*π*) based on cpDNA and nDNA dataset, were calculated in DNASP 6.0 (Librado & Rozas, 2009). The two indexes were also mapped using ‘ggplot2’ package in R 4.0.5 (https://cran.r-project.org/web/packages/ggplot2/index.html) (Wickham, 2016). Total genetic diversity across all populations (*H*_T_), average genetic diversity within populations (*H*_S_), and two parameters of population differentiation (*G*_ST_, *N*_ST_) were calculated using HAPLONST 3.0 (Pons & Petit, 1996). PERMUT 2.0 was used to test the significance of population differentiation (*G*_ST_, *N*_ST_) (Bandelt, Forster & Röhl, 1999; Pons & Petit, 1996) with 1,000 replicates. Phylogeographical structure is present if *N*_ST_ is significantly higher than *G*_ST_ (Pons & Petit, 1996).

An AMOVA analysis was employed to investigate genetic differentiation among overall populations and the defined population groups by phylogenetic clustering results using ARLEQUIN 3.5.2.2 (Excoffier & Lischer, 2010). The relationships among haplotypes were estimated using the median-joining method, implemented in Network 5.01 (Bandelt, Forster & Röhl, 1999). Based on the genetic distances among populations, we used Principal coordinate analyses (PCoAs) to analyze the genetic structure for *A. mongolica* using the ‘vegan’ package in R 4.0.5 (Oksanen et al., 2007), and then we performed 3D visualization using the ‘scatterplot3d’ package in R 4.0.5. Alleles In Space (Miller, 2005) was used to explore Genetic landscape shape analysis of *A. mongolica* which can reveal the level of genetic differentiation among different populations from the perspective of Landscape genetics, and the result of genetic landscape shape analysis plot which *x*- and *y*-axes correspond to geographical locations and the *z*-axis to genetic distance, and the peak height on the *z*-axis represents the degree of genetic differentiation between populations. Furthermore, based on Monmonier’s maximum difference algorithm, we estimated the possible genetic barriers in BARRIER v2.2 (Manni, Guérard & Heyer, 2004). We implemented a multiple matrices test based on 100 replicates of population average pairwise difference matrices in order to assess the robustness computed barriers. These matrices were generated by resampling of genotype sequences in DnaSP 6.0 (Librado & Rozas, 2009) and subsequent analyses in ARLEQUIN 3.5.2.2 (Excoffier & Lischer, 2010). In order to identify whether the genetic barriers and biogeographic barriers were spatially consistent, we corrected the possible spatial position of genetic barriers in ArcGIS 10.5 (ESRI, Redlands, CA, USA) according to the sampled population points.

### Phylogenetic analysis and estimation of divergence

Phylogenetic trees with cpDNA haplotypes and nDNA ribotypes in *A. mongolica* were reconstructed using maximum likelihood (ML) analysis with MEGA-X ver. 10.1.8 (Sudhir et al., 2018) and a Bayesian inference (BI), as implemented in BEAST 2.2.1 (Drummond & Rambaut, 2007) . *Amygdalus davidiana* and *Prunus triloba* were selected as the outgroups in the analysis owing to their relatively close evolutionary relationship (Delplancke et al., 2016). FigTree v1.4.4 (http://tree.bio.ed.ac.uk/software/figtree/) was used for tree-visualization (Zhao et al., 2019b).

The HKY substitution model was selected by MODELTEST as the best-fitting model for the data set (Posada & Crandall, 1998). Based on the synonymous substitution rates for most angiosperm species of cpDNA genes (1.0 × 10^−9^ to 3.0 × 10^−9^ s/s/y) (Wolfe, Li & Sharp, 1987), and substitution rates of plant ITS (3.46 × 10^−9^ to 8.69 × 10^−9^ s/s/y) (Richardson et al., 2001), we used a normal prior distribution and set a mean of 2 × 10^−9^ s/s/y and a standard deviation of 6.08 × 10^−10^ s/s/y for cpDNA, and a mean of 6.73 × 10^−9^ s/s/y and a standard deviation of 1.99 × 10^−9^ s/s/y for ITS (Zhang et al., 2013). The Markov chain Monte Carlo (MCMC) analysis was run for 10 million generations and sampled every 1000 generations for the Bayesian analysis. Bayes factor (BF) values were used to detect MCMC convergence, and the effective sample size (ESS) of each parameter above 200 after the first 10% of generations was discarded as burn-in (Baele et al., 2012).

### Demographic history analysis and potential migration corridors

The mismatch distribution analysis and the parameter calculations were all implemented in ARLEQUIN 3.5.2.2 (Rogers & Harpending, 1992). Tajima’s *D* (Tajima, 1989) and Fu’s *F*s statistics were calculated to test the recent demographic expansion. Significantly large negative values for *D* and *F*_S_ suggested that populations have experienced range expansion or natural selection (Fu, 1997; Tajima, 1989). And the Harpending’s raggedness index (*H*_rag_), the sum of squared deviation (*SSD*) and their *p* values were computed to test the significance of this population expansion model. A non-significant value (*p* > 0.05) of SSD and *H*_rag_ indicated population expansion (Excoffier, Laval & Schneider, 2005; Harpending, 1994). The mismatch distribution analysis was used to test whether *A. mongolica* underwent a recent range expansion for the overall populations and the defined groups (Excoffier & Lischer, 2010). Unimodal distributions of pairwise differences suggested populations that could have undergone range expansions, whereas populations in demographic equilibrium were characterized by multimodal mismatch distributions (Rogers & Harpending, 1992). Expansion tests with 10,000 permutations were performed for the significance test. To further detect the expansion trend and estimate the expansion time of *A. mongolica*, BEAST 2.2.1 was used to construct the Bayesian skyline plot (BSP) according to the previous sequence mutation rate in Bayesian inference, and Tracer 1.5 was used to generate the BSP (Rambaut & Drummond, 2009).

Based on the assumption that sites with higher species distribution frequencies have lower migration costs, the species distribution model was converted to a species habitat resistance model. We inverted the species distribution model (1-SDM) for the last glacial maximum (LGM, based on MIROC-ESM Model) and present (1970–2000) periods generated in MAXENT 3.4.1 to a “dispersal cost layer (resistance layer)”; Second, based on the least-cost path (LCP), the resistance layer of *A. mongolica* using SDMtoolbox 2.0 (Brown, 2014) in ArcGIS 10.5 was used to create a cost distance raster for each sample locality, and the corridor layers were established between every two localities based on the cost distance raster; Finally, all of the pairwise corridor layers were summed as the eventual dispersal corridor for *A. mongolica* (Jiang et al., 2018). The data required for species distribution model of LGM and present periods were obtained from our previous study (Ma et al., 2019).

### Identification of potential protection areas

Comprehensive consideration of population genetic diversity, the suitable distribution habitats and the Land use/cover were necessary for the scientific and effective conservation of species (Wei et al., 2014). The dataset of Genetic diversity landscapes, Land Use/Cover Change (LUCC) and the species distributions suitability of *A. mongolica* were employed to identify the potential conservation areas for *A. mongolica* (Wei, 2020). Genetic diversity was mapped by genetic landscape GIS toolbox in ArcGIS 10.5 according to genetic diversity values (*H*_d_ and *R*_d_) of each studied population and interpolated by using an established inverse-distance-weighted interpolation algorithm (Vandergast et al., 2011; Yu et al., 2014). LUCC (2018; 1 km × 1 km) in study area was obtained from the Data Center of Resources and Environment Sciences, Chinese Academy of Sciences (http://www.resdc.cn/). According to the characteristics of suitable habitats of *A. mongolica* (Ma et al., 2015), a correlation value was assigned to each different land use type of LUCC, and the suitable habitats (grassland, sand, bare rock, and others, etc.) were assigned values ranged from 0 to 1 (Table S3; Ren, 2019). Present potential suitable distribution data was obtained by species distribution models (MAXENT) from the previous study (Ma et al., 2019). The dataset of Genetic diversity landscapes, LUCC and the potential distribution were overlaid by using weight of 0.7, 0.2 and 0.1 in ArcGIS 10.5, and classified into three classes using the natural discontinuity grading method, which were defined as the low potential protection areas, medium potential protection areas and high potential protection areas (Wei, 2020).

### Environmental heterogeneity analysis of geographically weighted regression

Geographically weighted regression model (GWR) was implemented to test the relationship between genetic diversity values among *A. mongolica* populations and the environmental variables.

GWR was a spatial regression model that was an extension of traditional regression to predict, detect and estimate spatial non-stationary coefficient for model variables (Daniel, 2004), it can reflect the spatial heterogeneity of variable distribution (Abebaw et al., 2021).

We used the genetic diversity values (*H*_d_ and *R*_d_) of each population from both cpDNA and nDNA as the response variable. As predictor variables, we choosed 8 environmental factors from species distribution model and altitude data (Worldclim, http://www.worldclim.org), then we removed multicollinearity (variance inflation factor > 7.5) between variables independent variables, and finally we kept five factors which were altitude, annual mean temperature (Bio1), isothermality (Bio3), temperature annual range (Bio7), and mean temperature of driest quarter (Bio9). In order to obtain dependent variable, we used the fishing net tool generate points with resolution of 2,000 m, and then extracted the dependent variable and independent variable information in ArcGIS10.5. GWR4.08 (https://gwrtools.github.io/) software was used to finish geographically weighted regression analysis, and ARCGIS10.5 was used to analyze the results visually.

## Results

### The characteristics of cpDNA and nDNA sequences

The cpDNA aligned sequences of *trn*L-*trn*F, *psb*K-*psb*I and *trn*V were 402 bp, 230 bp and 572 bp in length respectively, and 1,204 bp in total. In the combined data (Table S1), we identified 17 different haplotypes (H1–H17) and 24 polymorphic sites (18 substitutions and 6 indels). The aligned fragment ITS1-ITS4 was 625 bp in length, with 9 nucleotide substitutions and 10 ribotypes (R1–R10) were defined (Table S2, Fig. 1).

### Haplotype/ribotypes geographical distributions

**Figure 2:**
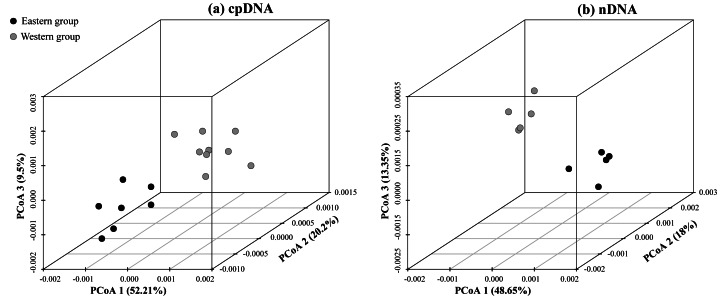
Plots of the first three coordinates of the principal coordinates analysis (PCoA) at a population level based on the cpDNA and nDNA pairwise differentiation matrix for *Amygdalus mongolica*. Colors of dots represent the individuals in the Western and Eastern groups (as in Fig. 1).

According to the cpDNA and nDNA dataset, the network analysis revealed two clades, the 9 haplotypes (H1–H9) and 5 ribotypes (R1–R5) in the Clade 1 are from the populations of the Western group, and the remaining 8 haplotypes (H10–H17) and 5 ribotypes (R6–R10) in the Clade 2 belong to the Eastern group, and no genotype was shared between the two groups (Fig. 1B). The results of PCoA show that the haplotypes and ribotypes mentioned above were independently clustered together, and the first three axes have explained a cumulative percent variation of 81.19% and 80%, respectively (Fig. 2).

The Western group mainly distribute in Alxa Left Banner and Hexi Corridor. The most widespread haplotype was H5, which shared by 4 populations and mainly distributed in Alxa Left Banner (ZQL, ZQK, WSH and WSM), and there are 7 private haplotypes (1, 2, 4, and 6–9) in the Western group. The most common ribotype was R3, which distributed among 5 populations, mainly from Hexi Corridor (YCQ) and Alxa Left Banner (ZTM, ZQL, ZTL and ZQK; Table 1 and Fig. 1).

In the Eastern group, the most widespread haplotype was H12, which were detected among each of the four populations and distributed in Urad Zhongqi (WLJ), Yinshan Mountains (YMM and YMF), and Urad Houqi (WLB); and five private haplotypes (10, 13, 14, 16 and 17) were revealed. The most common ribotype was R8, which distributed in Urad Zhongqi (WLJ), the Yinshan Mountains (YMF and YMM) and Urad Houqi (WLB); R7, R9 and R10 were detected as three private ribotypes (Table 1 and Fig. 1).

### Population genetic diversity and genetic differentiation

The cpDNA haplotype/nDNA ribotype diversity (*H*_d_, *R*_d_) among the 15 populations ranged from 0 to 0.567 (cpDNA) and 0 to 0.676 (nDNA); Nucleotide diversity (*π*) ranged from 0 to 0.00962 (cpDNA) and 0 to 0.00922 (nDNA), respectively (Table 1). According to both the cpDNA and nDNA datasets, the higher levels of genetic variation were identified in the populations MZS (Mazong Mountains), ZQK (Southwestern Lang Mountains) and WSH (Northern Helan Mountains) from the Western group, and the WLB (Urad Zhongqi) and BTQ (Daqing Mountains) from the Eastern group (Fig. S2).

**Figure 3:**
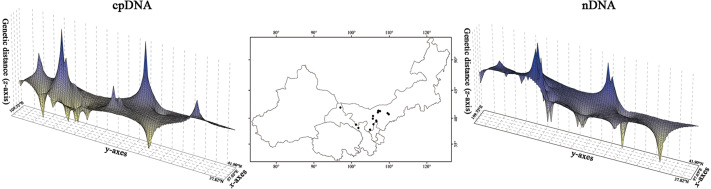
Spatial genetic landscapes constructed from the cpDNA sequences and nDNA sequences across the total distributions of *Amygdalus mongolica*. The abscissae and ordinates correspond to geographical coordinates covering the entire distributional populations, and the vertical axes represent genetic distances.

The *H*_T_ (cpDNA: 0.807 ± 0.050; nDNA: 0.815 ± 0.077) was much higher than *H*_S_ (cpDNA: 0.225 ± 0.053; nDNA: 0.212 ± 0.014) with both cpDNA and nDNA data, indicating considerable population differentiation. A higher level of *N*_ST_ (0.858/0.829; *P* < 0.05) than *G*_ST_ (0.712/0.690; *P* < 0.05), indicated a significant phylogeographical structure across the species range (Table 2). AMOVAs based on cpDNA and nDNA sequences showed that 83.87% and 79.33% of the total variation primarily occurred among populations, and 66.25% and 73.14% occurred among groups (Table 3). Results of PCoA showed that the populations from the Western and Eastern groups based on cpDNA and nDNA were independently clustered together, which revealed that the Western group was clearly distinct from the Eastern group (Fig. 2). The genetic landscape shape analysis of *A. mongolica* showed significantly divergence in study area, and signifcant genetic differentiation occurred between the Western and Eastern regions (Fig. 3). Meanwhile, two strong genetic barriers between the Western and Eastern groups, with over 81% bootstrap support, were detected in the Kubuqi and Ulanbuhe deserts based on Monmonier’s maximum difference algorithm (Figs. 1C, 1D).

**Table 2:** Estimates of gene diversity and population differentiation (mean ± SD) for the total populations of *Amygdalus mongolica*.

Data set	*H*_T_	*H*_S_	*G*_*ST*_	*N*_*ST*_
cpDNA	0.807 ± 0.050	0.225 ± 0.053	0.712 ± 0.070	0.858 ± 0.083
nDNA	0.815 ± 0.077	0.212 ± 0.014	0.690 ± 0.036	0.829 ± 0.013

**Notes.**

*H*_T_ total gene diversity *H*_S_ average gene diversity within populations *G*_*ST*_ and *N*_*ST*_ population differentiation values

**Table 3:** Analysis of molecular variance (AMOVA) for 15 populations of *Amygdalus mongolica*.

Data set	Source of variation	d. f.	Sum of squares	Variance components	Percentage of variation (%)	Fixation index
cpDNA	Among populations	14	612.031	4.356	83.87	
	Within populations	159	114.769	0.838	16.13	*F*_CT_ = 0.839
	Total	173	726.800	5.194		
	Among groups	5	421.824	2.862	66.25	*F*_SC_ = 0.731
	Among populations within groups	10	74.343	1.066	24.67	*F*_ST_ = 0.91
	Within populations	159	53.376	0.392	0.393	*F*_CT_ = 0.663
	Total	173	549.544	4.320		
nDNA	Among populations	14	163.713	1.166	79.33	
	Within populations	159	41.334	0.034	20.67	*F*_CT_ = 0.793
	Total	173	205.407	1.470		
	Among groups	5	149.993	1.116	73.14	*F*_SC_ = 0.314
	Among populations within groups	10	10.018	0.12	8.44	*F*_ST_ = 0.816
	Within populations	159	38.257	0.281	18.43	*F*_CT_ = 0.731
	Total	173	198.268	1.527		

**Notes.**

*F*_ST_ correlation within populations relative to the total *F*_SC_ correlation within populations relative to groups *F*_CT_ correlation of haplotypes/ ribotypes within groups relative to the total

### Phylogenetic analysis and divergence time dating

**Figure 4:**
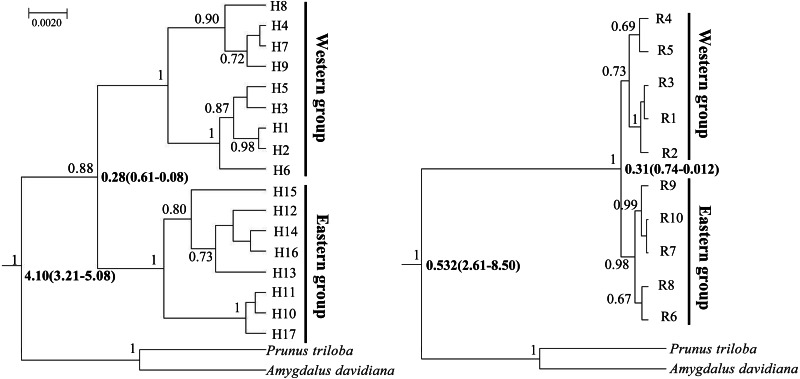
Bayesian divergence time estimates of *Amygdalus mongolica* based on 17 cpDNA haplotypes (left) and 10 nDNA ribotypes (right). The values above branches represent the posterior probabilities above 0.65 and the divergence time are show below branches.

Similar gene genealogies of genotypes in the BI trees and ML trees based on cpDNA and nDNA were obtained (Fig. 4 and Fig. S1). The nine haplotypes (1–9) and five ribotypes (1–5) corresponded to the Western group, while the remaining eight haplotypes (10–17) and five ribotypes (6–10) corresponded to the Eastern group (Fig. 4 and Fig. S1).

BEAST analysis showed that the cpDNA and nDNA divergence between the two groups of *A. mongolica* were 0.28 million years ago (Ma) and 0.31 Ma (Fig. 4). The timing of divergence for two groups are consistent with the middle Pleistocene during a period of strong expansion of aridification in northwestern China (Comes & Kadereit, 1998).

### Demographic history analysis

**Figure 5:**
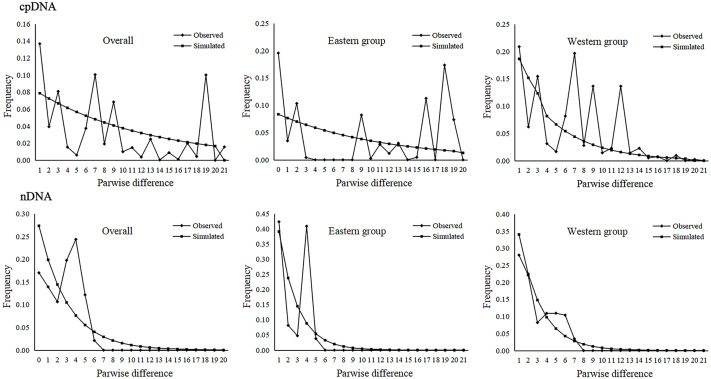
Mismatch distribution analysis based on the cpDNA and nDNA sequences for the total populations, as well as populations in the Eastern and Western groups (*i.e.*, the grouping of populations consistent with Fig. 1).

**Figure 6:**
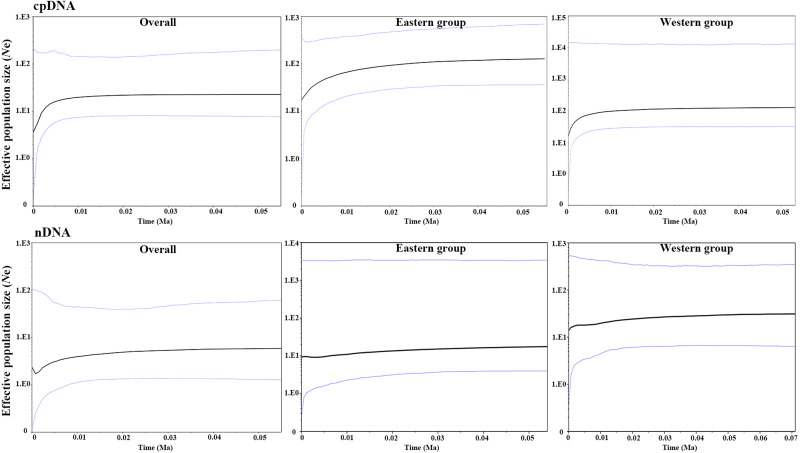
Bayesian skyline plot (BSP) estimating the time of *Amygdalus mongolica* population expansion based on the cpDNA and nDNA. The solid line indicates the median values, and the area between both purple lines represents the boundary of the 95% central posterior density interval. *X*-axis: time, years before present (Ma); *Y*-axis, effective population size (*N*e, the product of effective population size and generation length).

The parameters of Tajima’s *D* and Fu’s *Fs* for the overall population and the two groups were not significant and were positive, indicating that *A. mongolica* has not experienced a recent expansion (Table 4). Despite this, post-glacial range expansion indicated that the overall population as well as the two groups had evidence of recent expansion through the unimodal mismatch distribution curves of nDNA (Fig. 5). This conclusion was also supported by the *SSD* and *H*_*rag*_ which were not significant (*p* > 0.05) in cpDNA and nDNA for the overall populations or the two groups in cpDNA and nDNA (Table 4). However, the Bayesian skyline plot summarize instantaneous estimates of effective population size based on cpDNA for the total populations, the Eastern group, and the Western group, and showed recent population decline for *A. mongolica*. A slight population decline was found for the Eastern group and Western group, and a contraction followed by a small amount of population growth was found for the total population according to the nDNA (Fig. 6).

**Table 4:** Results of neutrality tests and mismatch distribution analysis for the overall populations and two regional groups based on the cpDNA and nDNA dataset.

Group	cpDNA dataset						nDNA dataset					
	SSD	*P*	*H*_rag_	P	Fu’s *F*s	Tajima’s *D*	SSD	*P*	*H*_rag_	P	Fu’s *F* s	Tajima’s *D*
Overall	0.184	0.072	0.337	0.391	2.365	1.3045	0.186	0.054	0.371	0.317	1.189	1.240
Eastern group	0.225	0.063	0.3	0.457	0.665	2.6346	0.197	0.245	0.351	0.354	2.734	1.154
Western group	0.091	0.126	0.335	0.303	6.495	−0.5173	0.191	0.079	0.397	0.297	1.142	1.020

**Notes.**

SSD sum of squared deviations *H*_*Rag*_ Harpending’s raggedness index

**Figure 7:**
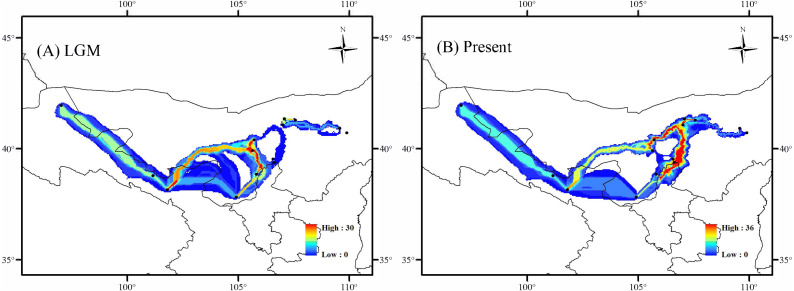
Dispersal corridors of *Amygdalus mongolica.* during (A) the last glacial maximum (LGM) period and (B) the present.

In the LGM based on MIROC-ESM climate model, the Lang Mountains, the edge of Tennger deserts, and the Hexi Corridor are the important corridors for the dispersal of *A. mongolica* (Fig. 7A). In the present period, the dispersal corridors of *A. mongolica* were similar to the model, but the edges of the Ulanbuhe deserts was identified as the most important migration corridors for the species (Fig. 7B).

### Identification of potential protection areas

**Figure 8:**
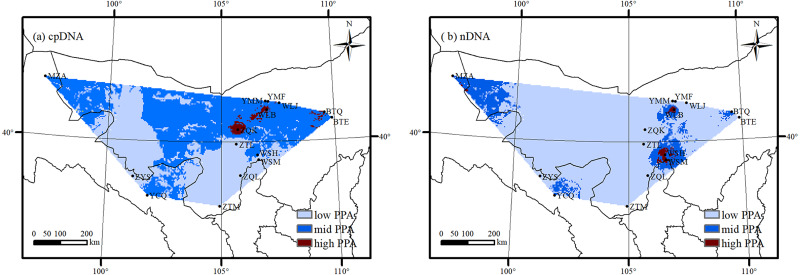
The potential protection area (PPA) of *Amygdalus mongolica* based on genetic diversity (A: cpDNA; B: nDNA).

Considering the overall genetic diversity, distribution suitability of *A. mongolica* and Land use/cover in northwestern China, high potential protection areas were identified in the south slopes of the Lang Mountains and the northern Helan Mountains (Fig. 8). These two areas had high genetic diversity based on cpDNA and nDNA (*H*_d_ and *R*_d_ >  0.70), high distribution suitability as determined by MAXENT (>0.80), and more natural habitats with little human activity (including sand, bare rock and grasslands).

### Environmental heterogeneity analysis

The spatial distribution of regression coefficients of different environmental factors was significantly different across in the study area, indicating spatial heterogeneity between different environmental factors and genetic diversity of *A. mongolica* (Figs. S4, S5). In the Eastern group region, genetic diversity was positively correlated with altitude, Bio1, Bio3, and Bio7, but was negatively correlated with Bio9. In the Western group region, genetic diversity was positively correlated with Bio1, Bio3, Bio7, and Bio9, but was negatively correlated with altitude (Figs. S4, S5).

## Discussion

### Allopatric divergence between the Western and Eastern regions

The strong phylogeographic structure of *A. mongolica* indicated that separate and isolated lineages occupy the different geographic regions (Fig. 1). Both the chloroplast and nuclear phylogenetic analyses showed two distinct lineages distributed in the Western and Eastern regions (Fig. 1). The network and PCoA analysis also indicated similar results: with nine haplotypes and five ribotypes from the Western region clustered together and apparently separated from the other seven haplotypes and five ribotypes distributed in the Eastern region (Figs. 1 and 2).

No incomplete genealogical sorting was detected within the distribution range of *A. mongolica*, indicating that the polymorphism of molecular markers used in this study were relatively appropriate. By contrast, we previously identified a large number of shared haplotypes (H2, 4, 6) between the Western and Eastern groups based on two cpDNA sequences, and phylogenetic analysis also revealed incomplete genealogical sorting (Ma et al., 2019). More than 75% of the observed differences were revealed between the two regions. The significant divergence among the Western and Eastern population groups was estimated to occur during mid-Pleistocene (Figs. 3 and 4). We therefore hypothesize that there is a close link between the increased aridification and desertification during the Quaternary glacial periods and intraspecific divergence of *A. mongolica*.

The mountains and deserts in the distribution areas of *A. mongolica* have leaded to distinct patterns of genetic isolation between 15 populations (Fig. 1), and these arid mountains slope and sand likely acted as geographical barriers and significantly affected interspecific differentiation of the desert land vegetation in northwestern China, such as *Euphrates poplar*, *Gymnocarpos przewalskii*, *Reaumuria soongarica* and *Lycium ruthenicum* (Jiang et al., 2018; Shi et al., 2020; Wang et al., 2021). For *A. mongolica*, the pre-existence and rapid expansions of the Ulanbuhe and Kubuqi deserts during Pleistocene (Guan et al., 2011), increased aridification and likely create potential barriers to gene flow between populations in the eastern and western regions. The two deserts were inferred as the genetic barriers for the two groups of *A. mongolica* (Figs. 1C, 1D).The sampled populations located in the different secondary valleys of the Yinshan Mountains, and the uplifts and eastward extensions of these valleys since the Quaternary period (Lee & Zhang, 2010), which likely further isolated the Eastern populations from the populations in the western regions. Moreover, in recent decades, with the intensification of drought and desertification in the arid northwestern China added the change of Land use/cover, *A. mongolica* and its natural habitats have been greatly disturbed and destroyed (Duan et al., 2020; Ma et al., 2015), the arid landscape fragmentation have caused the fragmented distributions and populations isolation, which hence restricted movement of pollen and seed of *A. mongolica*. Thus, the fragmentation of arid landscape was the potential barriers limiting gene flow, and it probably increased genetic differentiation among different populations. Furthermore, long-term isolation among plant populations in heterogeneous habitats can gradually lead to local adaptive differentiation and eventually create genetic heterogeneity across different landscapes (Chen et al., 2020; Jiang et al., 2018). For *A. mongolica*, the typical mountain basin–deserts isolation patterns between the eastern and western sampled areas, the arid landscape fragmentation and the corresponding environmental heterogeneity (Figs. S4, S5) have combined to promote differentiation between the Western-Eastern regions through local environmental adaptation.

### Pleistocene population history of *A. mongolica*

Climatic oscillations in the Quaternary period have caused range shifts for many xerophytic plants in the arid regions of northwestern China (Zhang, Zhang & Sanderson, 2016). During glacial periods, most desert plants reduced their distributions due to the unusually cold-dry climates, and had recolonized areas that are more suitable at the end of glacial periods (Sun et al., 2020; Zhao et al., 2019a). The unimodal mismatch distribution curves based on ITS sequences along with significant SSD and *H*_rag_ values all demonstrate that *A. mongolica* and the two population groups underwent post-glacial range expansions (Table 4 and Fig. 5). Considering the phylogenetic network according to cpDNA and nDNA datasets, genotypes H5/R3 and H12/R8 had high frequencies in the Western and Eastern groups, and “star-like” clusters originating from these genotypes mentioned were found (Fig. 1), also reflecting the historical signature of post-glacial expansions in the two regions. Plants in rapidly colonized regions generally possess low levels of genetic variation (Hewitt, 2000). For the Western group, the widespread distribution of H5 and R3 in the Alxa Left Banner (ZQK, WSH, WSM, ZQL, ZTM and ZTL) and the eastern Hexi Corridor (YCQ) supports the hypothesis that *A. mongolica* expanded westwards along the margins of the Ulanbuhe and Tengger deserts to the Hexi Corridor. Similarly, the demographic pattern of the widespread genotypes H12 and R8 revealed an expansionary trajectory of the Eastern group along the eastern edge of the Lang Mountains (Fig. 1). Moreover, the Bayesian skyline plot of species as determined by nDNA also identified a recent population expansion. Compared with cpDNA, the genetic information of nDNA can reflect the recent gene status. The low replacement rate of nDNA reduces the anti-mutation and parallel mutation, including the fixed differentiation information of a single locus and low convergence data to lead to better statistical results (Harris & Hey, 1999). The recent population expansion revealed by nDNA was likely associated with the warm and humid climate of the post-glacial period (Su & Zhang, 2013).

The Western and Eastern groups of *A. mongolica* experienced rapid range expansions during the Late Pleistocene-Early Holocene (Fig. 5). During the Late Pleistocene, the continued progress of aridification in northwestern China contributed to desert expansions, which in some cases may have provided a broader appropriate habitats (such as at the edges of deserts and arid piedmont grassland) for expansions of arid land plants (Ma, Zhang & Sanderson, 2012). The inferred post-glacial expansion tracks along desert margins have been revealed in some desert shrubs in northwestern China, including *Reaumuria soongarica*, *Nitraria tangutorum* and *Gymnocarpos przewalskii* (Jiang et al., 2018; Shi et al., 2020; Sun et al., 2020). For *A. mongolica*, the Western and Eastern groups were also found to have experienced significant outward expansions since the LGM, likely along the edges of the Ulanbuhe and Tengger deserts and Lang Mountains, respectively (Fig. 7). The warmer climates during the early Holocene facilitated snow melting on many of the peaks of the middle and south of the Qilian, Helan and Lang Mountains, a greater amount of runoff from melting snow and glacial ice resulted, making the local habitats of *A. mongolica*, such as the edges of the Ulanbuhe and Tengger deserts and the Lang Mountains wetter (Shi et al., 2003; Yang et al., 2011). The wet conditions favored the eastward migration of the eastern populations along the Lang Mountains and the westward migration of the western populations along the edges of the Ulanbuhe and Tengger deserts towards the Hexi Corridor.

### Conservation implications

Habitat fragmentation and reduction, such as increasing arable land area and urbanization, accompanied by low genetic variation, could ultimately increase the chance of genetic drift and inbreeding within populations. In this study, the arid landscape fragmentation led to the low levels of genetic variation within populations in the YMM (mid-levels of the Yinshan Mountains), YMF (foot of the Yinshan Mountains) and WLJ (Urad Zhongqi) in the Eastern group, and in the WSH (northern Helan Mountains), WSM (Wusutu town of Alxa Left Banner), and ZQL (Southern Helan Mountains) in the Western group (Fig. 1 and Table 1). An integrated assessment and identification of conservation centers is a more effective way to optimize conservation strategies for *A. mongolica* (Zhang, Li & Li, 2018). Overall, conservation populations with high levels of genetic diversity facilitate the maintenance of evolutionary potential and the potential for adaptation to future environment change (Zhang, Wang & Jia, 2020). However, it is important to consider habitat suitability and the current and future Land use/cover during establishment of potential protected areas. Because of this, the south slopes of the Lang Mountains and the northern Helan Mountains were identified as potential important protected areas, as they would be beneficial for accumulation of genetic variation, and generation of genetic mutations, ultimately increasing the evolutionary potential of *A. mongolica*. For these two protected areas, it is recommended that suitable strategies be adopted for *in situ* conservation, considering the habitats in both areas are relatively intact.

## Conclusions

This study suggests a combination of climatic fluctuations and aridification during the Quaternary period combined with significant environmental heterogeneity in the Western and Eastern groups to play an important role in the phylogeographic structure and genetic diversity of *A. mongolica*. For this species, the enhanced aridity along with expansions of the Ulanbuhe and Tengger deserts, and the eastward extension of the Yinshan Mountains since the Quaternary period likely interrupted gene flow, triggering the current divergence in the two allopatric regions. The south slopes of the Lang Mountains and northern Helan Mountains were identified as having the most potential to be important protected areas for *A. mongolica* which is meaningful for the management of this endangered species. Future experiments will focus on how to better quantitatively describe gene flow.

## Supplemental Information

10.7717/peerj.13345/supp-1 Supplemental Information 1 Maximum likelihood (ML) tree of *Amygdalus mongolica* based on 17 cpDNA haplotypes (left) and 10 nDNA ribotypes (right). Bootstrap values equal or larger than 0.60 are shown above branches Bootstrap values equal or larger than 0.60 are shown above branches.

10.7717/peerj.13345/supp-2 Supplemental Information 2 Haplotype diversity and nucleotide diversity for each of the studied population and within Western and Eastern geographic groups of *Amygdalus mongolica* based on the cpDNA (A) and nDNA sequences (B)

10.7717/peerj.13345/supp-3 Supplemental Information 3 Potential distribution based on MAXENT Nineteen bioclimatic variables data were derived from the World Climatic Database (http://www.worldclim.org). Through principal components analysis and Pearson correlation analysis, 8 remaining variables (out of the original 19) were used to model the species distributions in MAXENT 3.4.1.

10.7717/peerj.13345/supp-4 Supplemental Information 4 Regression coefficients of *Hd* and environmental factors in GWR

10.7717/peerj.13345/supp-5 Supplemental Information 5 Regression coefficients of *Rd* and environmental factors in GWR

10.7717/peerj.13345/supp-6 Supplemental Information 6 Variable nucleotide sites in three cpDNA sequences in 17 haplotypes of Amygdalus mongolica

10.7717/peerj.13345/supp-7 Supplemental Information 7 Variable nucleotide sites in nDNA sequences in 10 ribotypes of *Amygdalus mongolica*

10.7717/peerj.13345/supp-8 Supplemental Information 8 Suitability values of different land use in study area based on *Amygdalus mongolica*

## Additional Information and Declarations

### Data Availability

The following information was supplied regarding data availability: All haplotype and ribotypes sequences gathered in this study have been deposited in GenBank (*trnL-trnF*, MK248504 –MK248515; *psb*K-*psb*I, MK248492 –MK248503; *trn*V, MT387487 –MT387503; ITS1- ITS4, MT397024 –MT397033). The accession numbers of the outgroup sequences for haplotype and ribotypes of *Amygdalus davidiana* are MK798145.1 and MH711207.1. The accession numbers of the outgroup sequences for haplotype and ribotypes of *Prunus triloba* are MK790138.1 and EU669088.1, respectively.
