# Supplementary figures and images for: Phylogeography of Amygdalus mongolica in relation to Quaternary climatic aridification and oscillations in northwestern China
Source: PeerJ. 2022 Apr 29;10:e13345. doi: 10.7717/peerj.13345 (PMC9059755; doi:10.7717/peerj.13345)

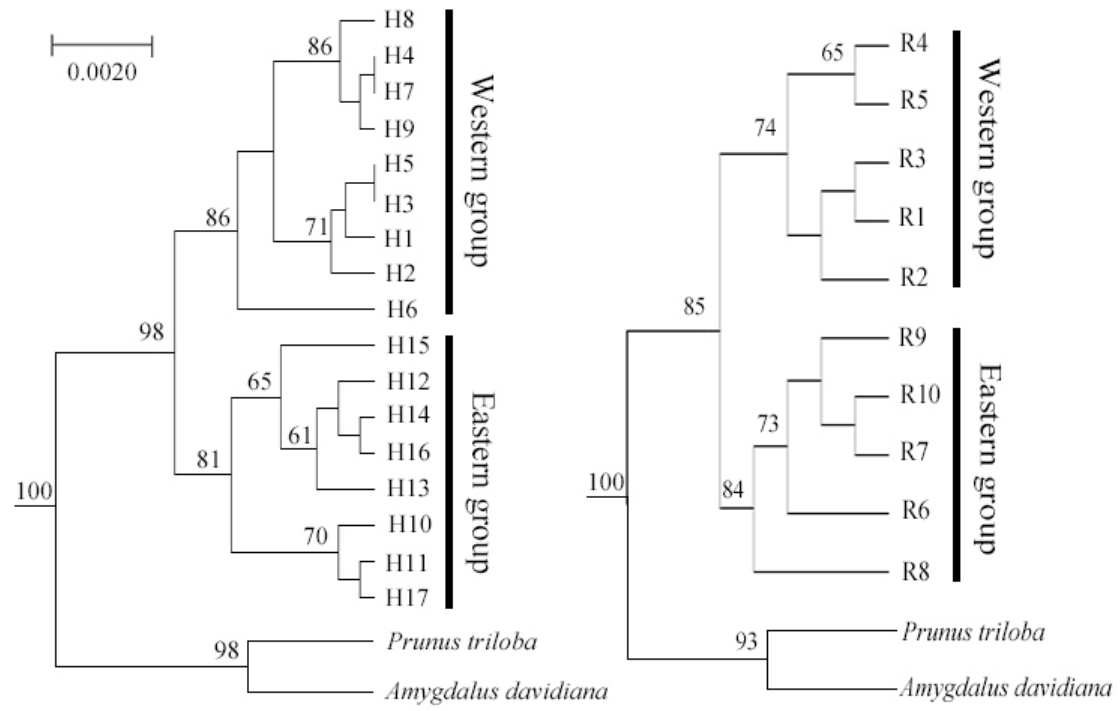

Supplement: Supplemental Information 1 — Bootstrap values equal or larger than 0.60 are shown above branches. [file peerj-10-13345-s001.pdf]

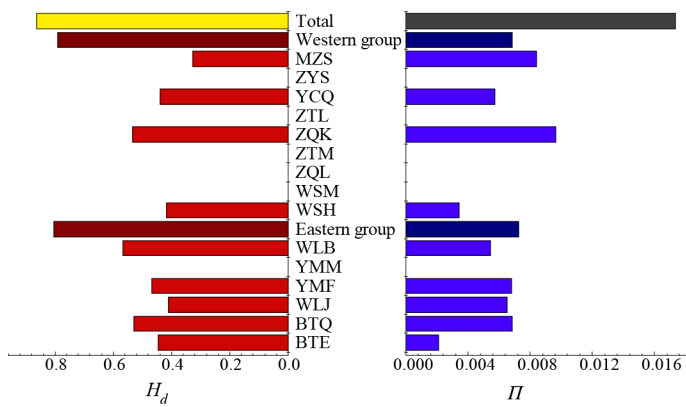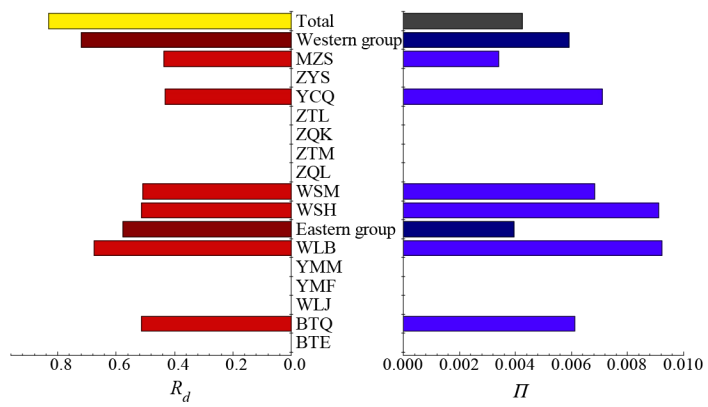

Supplement: Supplemental Information 2 [file peerj-10-13345-s002.pdf]

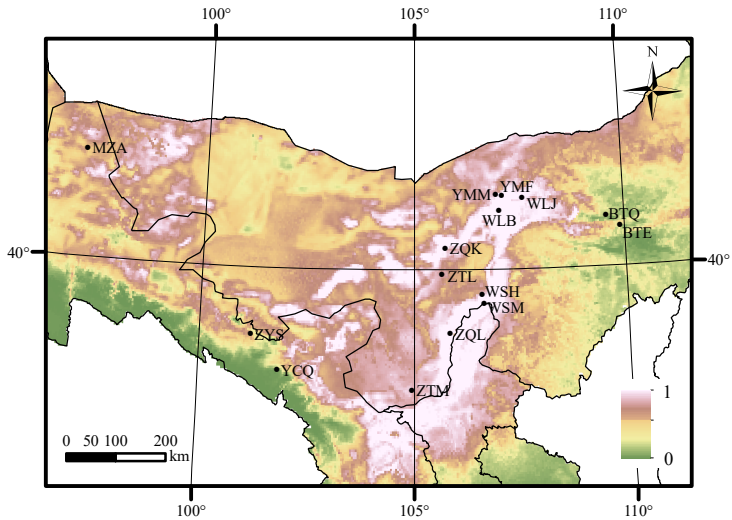

Supplement: Supplemental Information 3 — Nineteen bioclimatic variables data were derived from the World Climatic Database (http://www.worldclim.org). Through principal components analysis and Pearson correlation analysis, 8 remaining variables (out of the original 19) were used to model the species distributions in MAXENT 3.4.1. [file peerj-10-13345-s003.pdf]

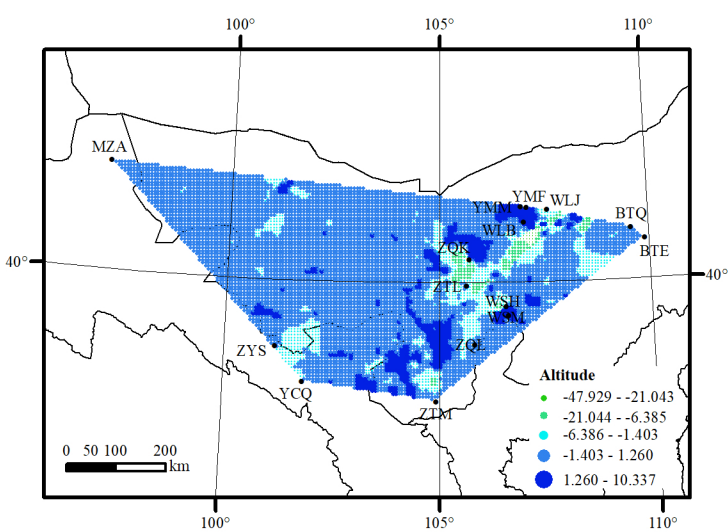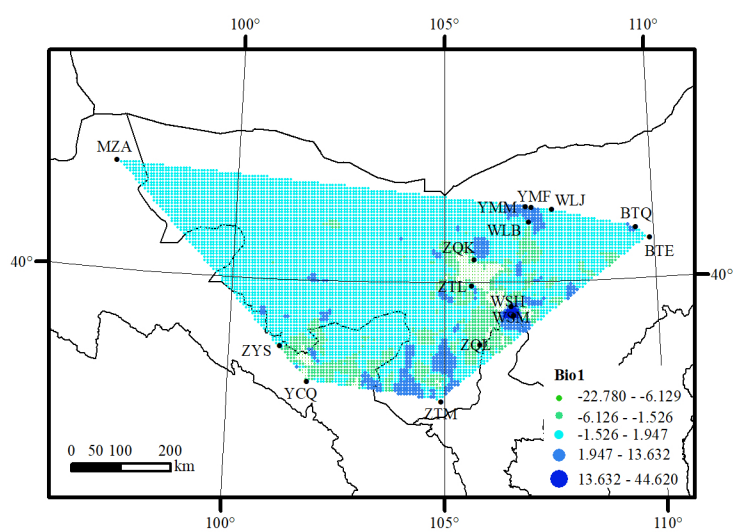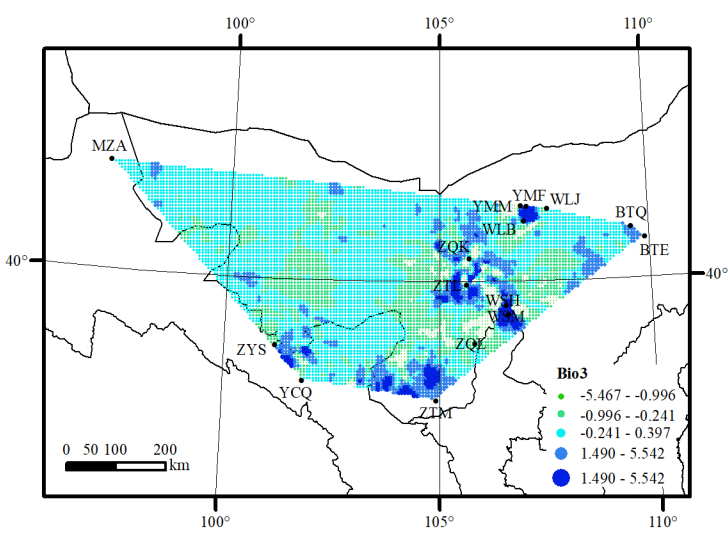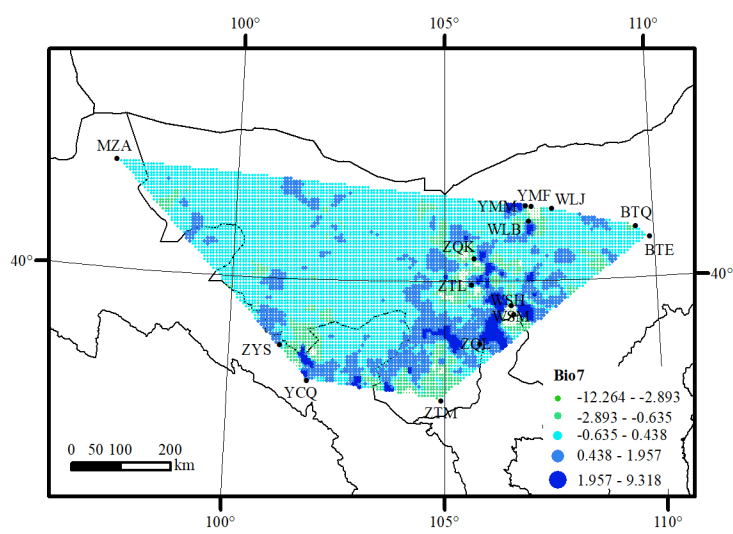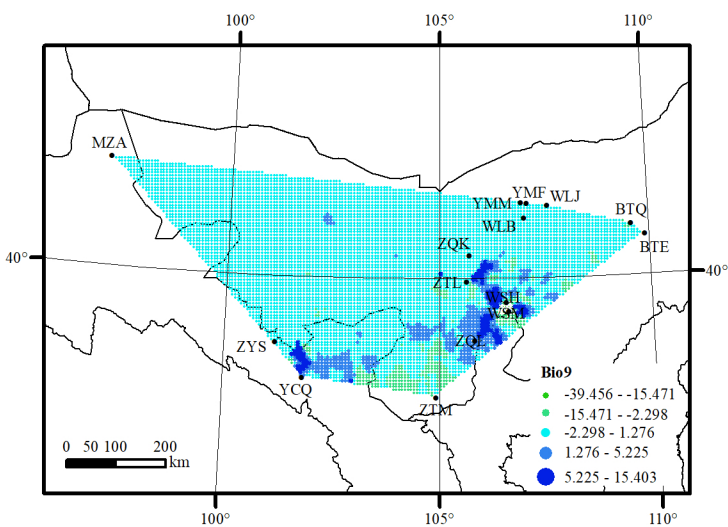

Supplement: Supplemental Information 4 [file peerj-10-13345-s004.pdf]

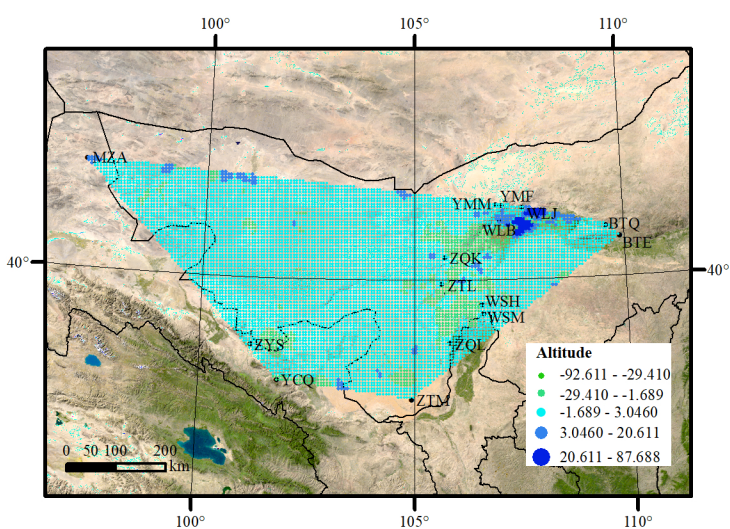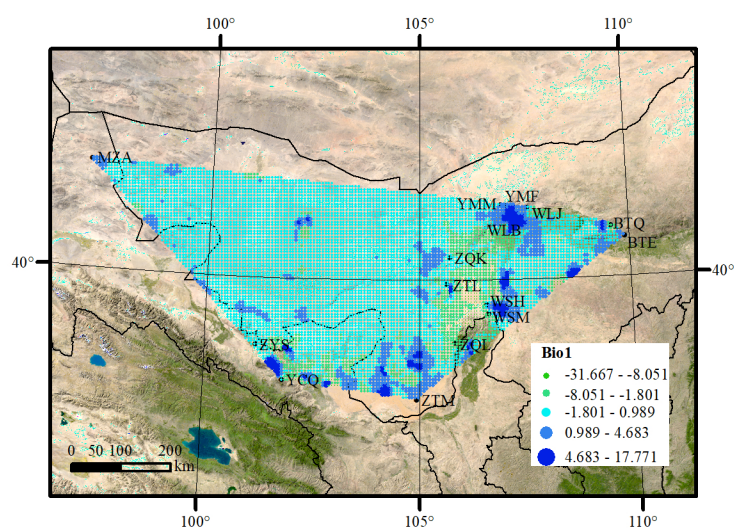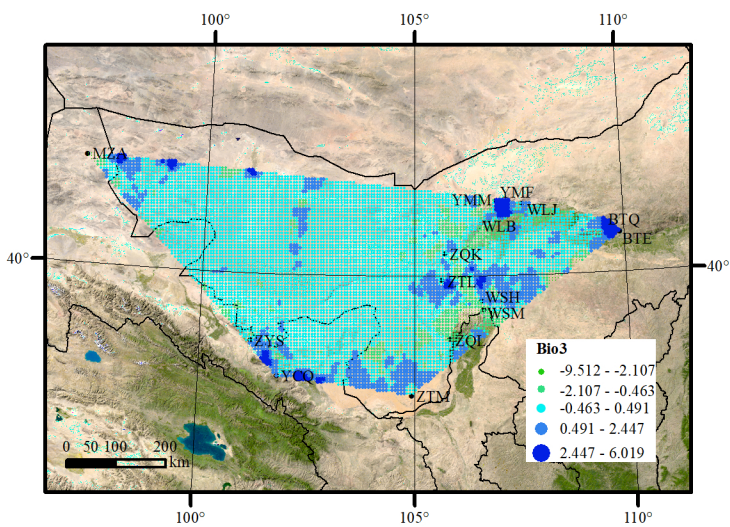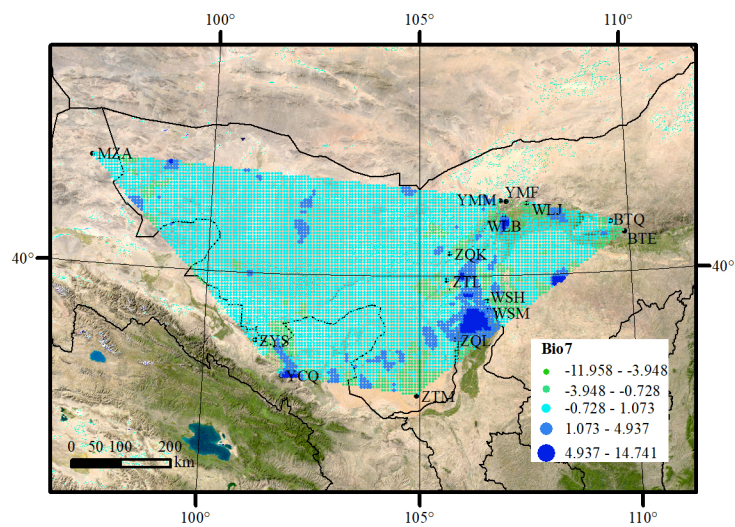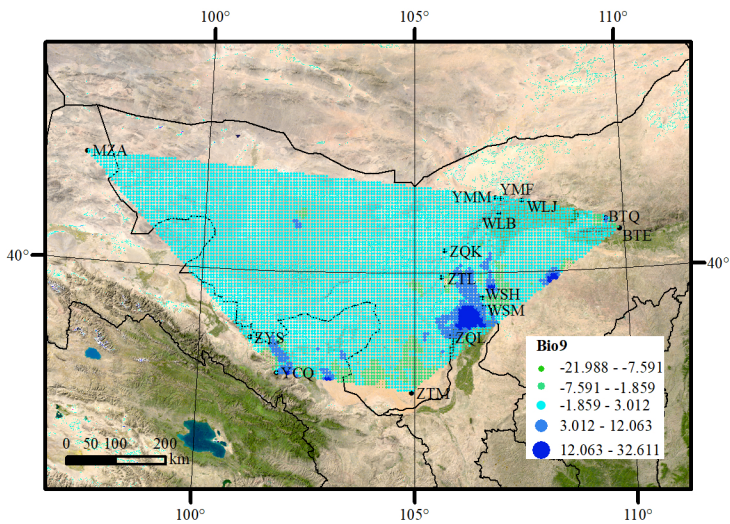

Supplement: Supplemental Information 5 [file peerj-10-13345-s005.pdf]
