# Supplementary material for: Phylogeography of Amygdalus mongolica in relation to Quaternary climatic aridification and oscillations in northwestern China
Source: PeerJ. 2022 Apr 29;10:e13345. doi: 10.7717/peerj.13345 (PMC9059755; doi:10.7717/peerj.13345)
Supplement: Supplemental Information 6 [file peerj-10-13345-s006.docx]

**Table S1** Variable nucleotide sites in three cpDNA sequences in 17 haplotypes of *Amygdalus mongolica*

| Haplotype | Variable nucleotide sites | | | | | | | | | | | | | | | | | | | | | | | |
| --- | --- | --- | --- | --- | --- | --- | --- | --- | --- | --- | --- | --- | --- | --- | --- | --- | --- | --- | --- | --- | --- | --- | --- | --- |
|  | *trn*L-*trn*F | | | | | | | | | | | *psb*K-*psb*I | | | | | *trn*V | | | | | | | |
|  | 3  6 | 50 | 92 | 106 | 115 | 189 | 223 | 290 | 338 | 345 | 357 | 23 | 142 | 143 | 146 | 147 | 8 | 107 | 112 | 178 | 191 | 229 | 327 | 5  4  6 |
| H1 | C | ■ | G | ▲ | ○ | ▼ | C | G | ◆ | G | A | ★ | - | - | - | - | A | G | C | T | C | C | G | A |
| H2 | . | □ | . | ▲ | ○ | ▽ | . | . | ◆ | . | . | ★ | . | . | . | . | . | . | . | . | . | . | . | A |
| H3 | A | □ | . | ▲ | ○ | ▽ | . | . | ◆ | . | . | ★ | . | . | . | . | . | . | . | . | . | . | . | A |
| H4 | . | □ | . | ▲ | ○ | ▽ | . | . | ◆ | . | . | ☆ | . | . | . | . | . | . | . | . | . | . | . | - |
| H5 | . | □ | . | △ | ○ | ▽ | . | . | ◇ | . | . | ★ | . | T | . | . | . | . | . | . | . | . | . | . |
| H6 | . | □ | . | ▲ | ○ | ▽ | . | . | ◆ | . | . | ★ | . | T | . | . | . | . | . | . | . | . | . | . |
| H7 | . | □ | . | ▲ | ○ | ▼ | . | . | ◆ | . | . | ★ | T | . | . | . | . | . |  |  | . |  | T | . |
| H8 | . | □ | . | ▲ | ○ | ▼ | . | . | ◆ | . | . | ★ | T | . | . | . | . | . | . |  | . |  | T | . |
| H9 | . | □ | . | ▲ | ○ | ▼ | . | . | ◆ | . | . | ★ | T | . | . | . | . | . | . | . | . | . | . | . |
| H10 | . | □ | . | △ | ○ | ▽ | A | A | ◇ | A | . | ★ | . | . | . | . | . | . | . | . | . | . | . | . |
| H11 | . | ■ | . | △ | ○ | ▽ | . | . | ◇ | . | - | ★ | . | . | . | . | . | . | . | . | . |  | . | A |
| H12 | . | ■ | . | △ | ○ | ▽ | . | . | ◇ | . | . | ★ | T |  | T | T | . | A | . | . | . | T | . | . |
| H13 | . | □ | T | △ | ○ | ▽ | . | . | ◇ | . | . | ★ | . | . | . | . | . | . | . | . | . | T | . | . |
| H14 | . | ■ | . | △ | ○ | ▽ | . | . | ◇ | . | . | ★ | . | . | . | . | . | . | . | . | T |  | T | . |
| H15 | . | □ | T | △ | ○ | ▽ | . | . | ◇ | . | . | ★ | . | . | . | . | . | . | . | . | . |  | . | . |
| H16 | . | □ | . | ▲ | ○ | ▼ | . | . | ◆ | . | . | ☆ | T | . | . | . | . | . | T | . | . | T | . | . |
| H17 | . | □ | . | ▲ | ○ | ▼ | . | . | ◆ | . | . | ★ | . | . | . | . | C | . | . | G | . | T | . | . |

■, TTATTTT; ▲, TATTAC; ●, TACAAA; ▼, CAAGT; ◆, AAAGA; ★, TTTTTA; ◢TTTTTA. Open symbols, i. e. □, △, ○, ▽, ◇, ☆ and ⊿ represent the indels format text, with the length >1 bp. ‘–’ represent indels, with the length equal to 1 bp
