# Supplementary material for: Phylogeography of Amygdalus mongolica in relation to Quaternary climatic aridification and oscillations in northwestern China
Source: PeerJ. 2022 Apr 29;10:e13345. doi: 10.7717/peerj.13345 (PMC9059755; doi:10.7717/peerj.13345)
Supplement: Supplemental Information 7 [file peerj-10-13345-s007.docx]

**Table S2** Variable nucleotide sites in nDNA sequences in 10 ribotypes of *Amygdalus mongolica*

| Ribotype | Variable nucleotide sites | | | | | | | | |
| --- | --- | --- | --- | --- | --- | --- | --- | --- | --- |
|  | ITS1- ITS4 | | | | | | | | |
|  | 90 | 273 | 309 | 425 | 431 | 457 | 519 | 573 | 583 |
| R1 | C | G | G | T | C | G | G | T | C |
| R2 | T | . | . | . | . | . | . | . | T |
| R3 | . | A | A | G | . | . | . | . | . |
| R4 | . | . | . | G | G | . | . | . | . |
| R5 | . | A | A | G | . | . | A | . | . |
| R6 | T | A | A | G | . | . | . | . | . |
| R7 | . | . | . | G | . | . | . | . | . |
| R8 | . | . | . | . | . | C | . | . | T |
| R9 | . | . | . | . | . | . | . | A | T |
| R10 | G | . | . | . | . | . | . | . | . |
