# Supplementary material for: Phylogeography of Amygdalus mongolica in relation to Quaternary climatic aridification and oscillations in northwestern China
Source: PeerJ. 2022 Apr 29;10:e13345. doi: 10.7717/peerj.13345 (PMC9059755; doi:10.7717/peerj.13345)
Supplement: Supplemental Information 8 [file peerj-10-13345-s008.docx]

**Table S3** Suitability values of different land use in study area based on *Amygdalus mongolica* habitat characteristics

| Land use type | [Evaluation](C:/Users/Administrator/AppData/Local/youdao/dict/Application/8.9.3.0/resultui/html/index.html#/javascript:;) |
| --- | --- |
| Sand, Bare rock | 1 |
| Gobi Desert | 0.8 |
| Grassland | 0.4 |
| Saline and alkaline land | 0.2 |
| else | 0 |
